# Recommendations on priorities for integrated palliative care: transparent expert consultation with international leaders for the InSuP-C project

**DOI:** 10.1186/s12904-019-0418-5

**Published:** 2019-04-03

**Authors:** Sheila Payne, Sean Hughes, Joann Wilkinson, Jeroen Hasselaar, Nancy Preston

**Affiliations:** 1 International Observatory on End of Life Care, Division of Health Research, Lancaster University, Lancaster, LA1 4YG UK; 2 Department of Anaesthesiology, Pain and Palliative Medicine, Radboud University Medical Center, Nijmegen, The Netherlands

**Keywords:** Consensus, Integrated, Palliative care, International survey

## Abstract

**Background:**

The World Health Organisation (WHO) endorses integrated palliative care which has a significant impact on quality of life and satisfaction with care. Effective integration between hospices, palliative care services, hospitals and primary care services are required to support patients with palliative care needs. Studies have indicated that little is known about which aspects are regarded as most important and should be priorities for international implementation. The Integrated Palliative Care in cancer and chronic conditions (InSup-C) project, aimed to investigate integrated practices in Europe and to formulate requirements for effective palliative care integration. It aimed to develop recommendations, and to agree priorities, for integrated palliative care linked to the InSuP-C project.

**Methods:**

Transparent expert consultation was adopted at the approach used. Data were collected in two phases: 1) international transparent expert consultation using face-to-face roundtable discussions at a one day workshop in Brussels, and 2) via subsequent online cross-sectional survey where items were rated to indicate degree of agreement on their importance and ranked to indicate priority for implementation. Workshop discussions used content analysis to develop a list of 23 recommendations, which formed the survey questionnaire. Survey analysis used descriptive statistics and qualitative content analysis of open responses.

**Results:**

Thirty-six international experts in palliative care and cancer care, including senior clinicians, researchers, leaders of relevant international organisations and funders, were invited to a face-to-face workshop. Data were collected from 33 (19 men, 14 women), 3 declined. They mostly came from European countries (31), USA (1) and Australia (1). Twenty one of them also completed the subsequent online survey (response rate 63%). We generated 23 written statements that were grouped into the organisational constructs: macro (10), meso (6) and micro (7) levels of integration of palliative care. Highest priority recommendations refer to education, leadership and policy-making, medium priority recommendations focused on funding and relationship-building, and lower priority recommendations related to improving systems and infrastructure.

**Conclusions:**

Our findings suggest that amongst a group of international experts there was overall good agreement on the importance of recommendations for integrated palliative care. Understanding expert’s priorities is important and can guide practice, policymaking and future research.

## Background

Healthcare today ideally seeks to offer an integrated service where patients’ and their families’ needs are addressed seamlessly across health and social care providers, and by different disciplines. Integrated palliative care improves quality of life, service coordination, efficiency and satisfaction with care [1]. Moreover, the World Health Assembly (WHA) [2] advocated that governments integrate palliative care into national health care systems across the life span. However, evidence suggests that the majority of the world’s population do not have access to any palliative care, let alone services that are integrated within national healthcare systems [3]. There is little agreement on which aspects of integration are important and which should be prioritised. This study aimed to provide an international consensus on recommendations and identify priorities for the implementation of integrated palliative care.

A European study, called InSuP-C, described integrated palliative care as bringing together administrative, organisational, clinical and service elements in order to ensure continuity of care delivered by all health and social care sectors involved in the care network of patients receiving palliative care [4]. A typology of integrated palliative care was developed to guide the implementation of integrated care [5]. Integration of palliative care may occur at three levels:

- Macro - incorporation of palliative care into national health care strategies and resource allocation plans
- Meso - inclusion of palliative care into regional, local and organisational health care services
- Micro - working at the level of specific patients and families, ensuring that palliative care operates in association with other medical disciplines such as oncology, neurology and geriatrics so that patients experience seamless care [6].

The European InSuP-C study on patient-centered integrated palliative care pathways in advanced cancer, chronic heart disease and Chronic Obstructive Pulmonary Disease (COPD) showed evidence of the limited development of integrated care for patients with heart failure and COPD compared to those with advanced cancer [4, 6, 7]. Evidence from analysis of 19 European integrated palliative care initiatives demonstrated that enhancing professional education, referral pathways and guidelines, and improving information exchange are key determinants that foster integration [8]. Across Europe, integrated palliative care initiatives have been identified, with large programmes implemented in some countries including Spain, Scotland, England, The Netherlands, France and Belgium [9]. Most initiatives seek to improve the early identification of patients with palliative care needs, enhance access to essential medicines, provide domiciliary nursing care, especially at night and near the end of life, and to increase the knowledge and skills of general practitioners and home care nurses. Gomez and colleagues made 10 recommendation to integrate a palliative care approach more fully into health and social care services [9]. However, policy makers, funders and health professionals may not share similar understandings and they may need support in identifying priorities for the implementation of integrated palliative care.

## Methods

### Aim

The aim of this paper is to investigate the content and the degree of consensus between palliative care experts about key recommendations for the further integration of palliative care at a micro, a meso and a macro level.

The outcomes reported in this paper were part of establishing valid international recommendations from the InSuP-C project. This original project used multiple embedded case study methods that aimed to identify factors associated with ‘good practice’ in 23 integrated palliative care initiatives in advanced cancer, heart failure and COPD in five European countries [4]. The protocol and reports are available [4, 6–8].

### Design of the Study

A two-phase consensus building process was undertaken over a 3 month period (September – December 2016) which involved two phases: 1) international expert consultation using face-to-face roundtable discussions, which generated written statements on macro, meso and micro organisational levels of integration of palliative care, and 2) a follow-up online cross-sectional survey where items were rated to indicate degree of agreement and ranked to indicate priority for implementation. The study design was informed by the MORECare Transparent Expert Consultation (TEC) approach to conducting a consultation workshop and roundtable discussions with experts in palliative care research [10]. TEC is a rapid means to elicit recommendations for action, using nominal group techniques to generate them, and an online survey for ranking to ascertain consensus [10]. This work aimed to: 1) generate consensus on recommendations for integrated palliative care, and 2) determine which recommendations are regarded as priorities for implementation.

### Setting and participants

Phase 1 of the study was conducted with international experts in palliative and cancer care at a face-to-face roundtable workshop held in Brussels on 29th September 2016. We defined integrated palliative care using the typology previously generated [5]. We established a panel of experts who were opinion drivers including international leaders, researchers and clinicians in palliative care, cancer care, specialists in chronic disease management including heart disease and COPD, leaders of relevant NGOs/INGOs such as the World Health Organisation, and relevant international funders. The international experts were identified through their relevant publications and searches on the internet. Experts were invited by email and their travel expenses were covered but no other incentives provided. In Phase 2, the workshop participants were invited to respond to an online survey by 30th November 2016.

### Data collection

Phase 1: The purpose of the consultative workshop was to draw upon the findings and three systematic reviews linked to the InSuP-C project [6, 11–13]; to discuss the implications for implementation in different socio-political, cultural and economic environments, and to develop strategic recommendations. The agenda was designed to present an overview of the project and introduce project results at three levels: macro, meso and micro [6]. The focus of the workshop was on participation and drawing on the expertise and professional knowledge of participants. Three concurrent groups were organised using nominal group techniques [14]. Groups were facilitated to provide an opportunity for all participants to make a contribution and an observer recorded detailed notes.

Phase 2: The 23 statements generated in Phase 1 were prepared as an online survey using Survey Monkey with a covering invitation email. The recommendations were presented in random order, and were attributed to one of three categories:

- macro – national/international level,
- meso – organisational/institutional level,
- micro – interactions between patients, families and health and social care professionals.

Participants were invited to rate the priority for implementation of each item using a Likert scale of 0–9 (where 0 indicated lowest and 9 indicating highest priority), and to rank all items relative to each other. Open comments on the items were possible. Responses were anonymised and one reminder was sent.

### Data analysis

Phase 1: All workshop discussion group notes were transcribed. All data were systematically compared and discussed by the co-authors (SP, NP) to ensure adequate synthesis of similarities and differences in the views expressed.

Phase 2: We report descriptive statistics for the survey items. For each statement, we report median agreement to determine the highest ranked items and interquartile (IQ) and total range to determine the degree of consensus. Respondents made very few narrative comments, but these helped to clarify recommendations.

The two-phases of activity reported in this paper were undertaken as part of the dissemination strategy of the InSuP-C project. As such, we did not seek formal research ethics approval, as this was not required as a dissemination activity in The Netherlands where the project was based, and is congruent with other published TEC studies [15]. However, we informed participants in writing prior to the workshop, and again prior to the survey, that their anonymised contributions would be used to develop recommendations, which would be distributed via an online survey, and that outcomes from both phases would be subsequently published. Thus, their involvement in both activities were regarded as implied consent.

## Results

In total, 33 people attended the workshop. Their characteristics are shown in Table 1. There were more men (*n* = 19) than women (*n* = 14). They came from 11 mostly European countries, with over representation from the Netherlands (9) and UK (7), and outside Europe, USA (1) and Australia (1). The majority held clinical and/or research roles (19) or represented NGOs/INGOs (11). Three people declined the invitation, as they were unavailable to attend the workshop. For the online survey, there were 21 respondents, a response rate of 63%.

**Table 1:** Characteristics of attendees at consultative workshop

Participant Number	Gender	Country	Role/Expertise
1.	Male	France	Clinician
2.	Female	Switzerland	INGO
3.	Female	United Kingdom	Manager
4.	Male	Spain	Researcher/clinician
5.	Male	USA	INGO
6.	Female	Hungary	Clinician
7.	Male	Australia	Policy maker
8.	Female	Greece	Clinician
9.	Female	United Kingdom	NGO/funder
10.	Male	Belgium	Researcher
11.	Male	United Kingdom	NGO
12.	Male	Spain	Researcher
13.	Male	Spain	Clinician/Researcher
14.	Male	The Netherlands	Researcher
15.	Male	United Kingdom	Researcher
16.	Male	Ireland	INGO
17.	Female	Ireland	INGO
18.	Male	Belgium	Clinician
19.	Male	United Kingdom	NGO
20.	Female	Switzerland	INGO
21.	Female	United Kingdom	Researcher
22.	Male	The Netherlands	INGO
23.	Female	The Netherlands	Clinician
24.	Male	Poland	INGO
25.	Female	The Netherlands	Volunteer
26.	Female	Belgium	INGO
27.	Female	Belgium	Clinician/researcher
28.	Female	The Netherlands	Researcher
29.	Male	The Netherlands	Researcher
30.	Male	The Netherlands	Researcher
31.	Male	The Netherlands	Researcher
32.	Female	United Kingdom	Researcher
33.	Male	The Netherlands	Clinician/researcher

Analysis of workshop discussions resulted in 23 statements on integrated palliative care. These referred to a range of palliative care topics including, education, awareness-raising, leadership, policy-making, ensuring quality of care, relationship-building, improving systems and infrastructure, and funding. The majority of recommendations concerned national or international levels (macro *n* = 10), with six focusing on the institutional level (meso) and with seven focusing on clinical interactions between patients, families and health professionals (micro) (see Table 2).

**Table 2:** Recommendations for Integrated Palliative Care at macro, meso and micro levels, presented in rank order

Ranking	Recommendation	Macro	Meso	Micro	Item No.
1	Palliative care should be integrated into mandatory education for undergraduate medical, health and social care professionals.	✓			12
2	Outcome measures to assess quality of integrated palliative care services should be developed.		✓		1
3	The digital transfer of information should be integrated within and across different palliative care services and general services including community and hospital teams, and patients and families.		✓		3
4	National palliative care regulations and policies should be extended to apply to all patients with palliative care needs, not just those with cancer.	✓			5
5	Clarification of the language and terms used to describe integrated palliative care and associated services is needed.	✓			10
6	There is a need for strong leadership to advocate for integrated palliative care.	✓			16
7	Raise awareness of integrated palliative care for senior managers and policy makers.		✓		20
8	Disease/condition specific national policies should integrate palliative care.	✓			15
9	Continuing professional development for all health and social care professionals should include coverage of integrated palliative care.	✓			13
10	For integration to work, new and creative ways of securing resources and specific funding should be established which can support the palliative care infrastructure.	✓			4
11	There needs to be national level strategic lobbying to develop and fund better integrated palliative care.	✓			9
12	Develop alliances within and between sectors to build better integration.		✓		11
13	Social care should be part of integrated palliative care.	✓			14
14	Establish needs based referral systems to guide timely referrals to integrated palliative care.			✓	18
15	Outcomes of integrated palliative care should be audited and benchmarked.		✓		22
16	Building of informal relationships are a foundation for formal structures which are pivotal for the integration of palliative care.			✓	6
17	Clinical protocols should be introduced to ensure integration of services for patients and families regardless of the setting where they are treated.			✓	7
18	Develop systems that provide adequate out-of-hours palliative care so that health care practitioners can maintain their work/life balance.			✓	8
19	Access to readily available and affordable essential medicines are necessary for integrated palliative care.			✓	21
20	An information hub (online or face-to-face) with a care co-ordination team should be established to contribute to the integration of palliative care services across the area.		✓		2
21	There is a need to invest in the development of future palliative care leadership skills.	✓			17
22	Establish a single point of contact for integrated palliative care at local level.			✓	19
23	Raise public awareness about palliative care and its integration with healthcare.			✓	23
		10	6	7	

The recommendations were attributed to three levels:

• macro – national/international level

• meso – organisational/institutional level

• micro – interactions between patients, families and health and social care professionals

### Prioritisation of recommendations

**Fig. 1:**
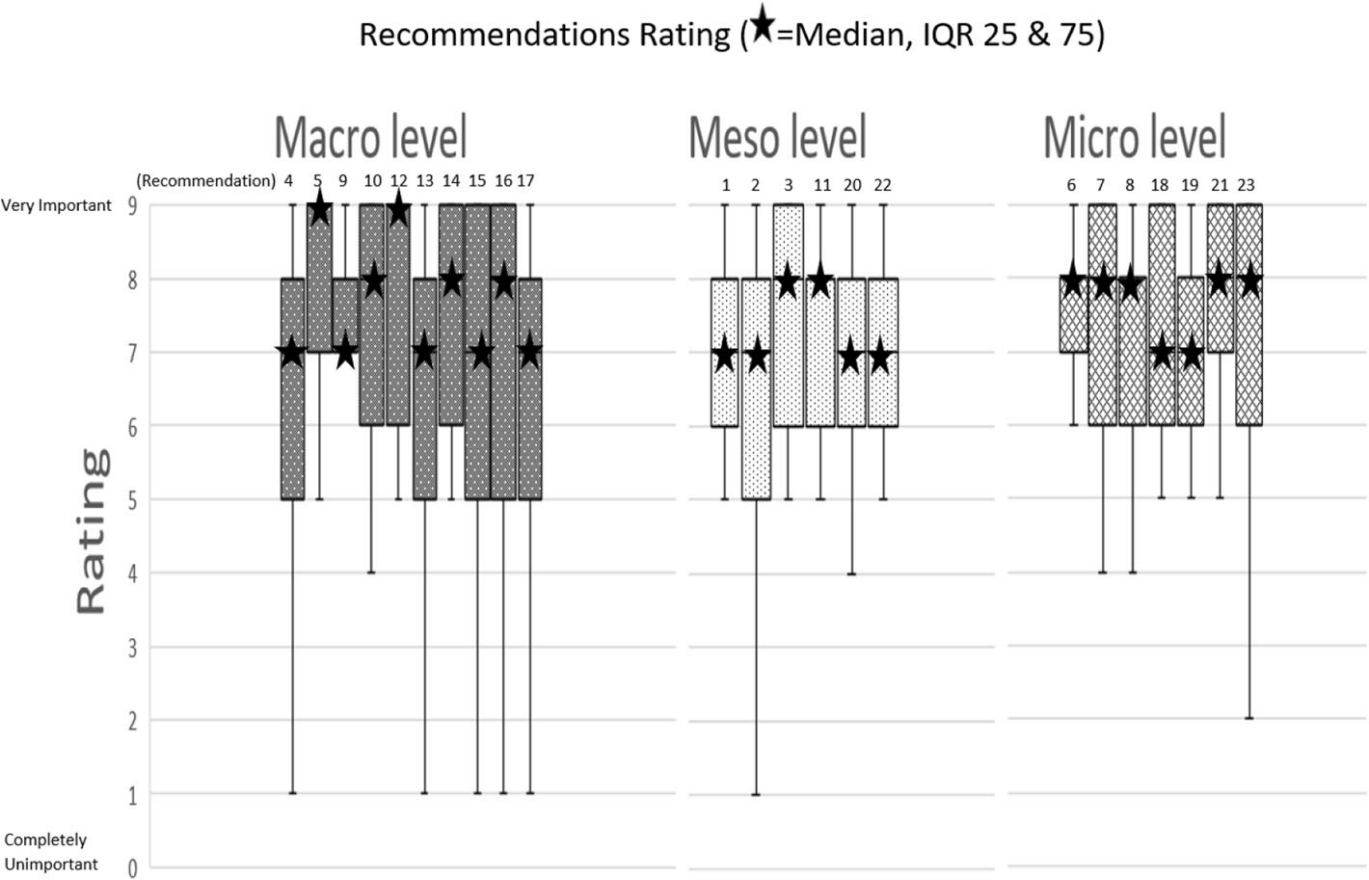
Medians and IQR of recommendation statements in three categories. The recommendations were attributed to three categories: • macro–national/international level, • meso–organisational/institutional level, • micro–interactions between patients, families and health and social care professionals

Following the survey, analysis of the degree of consensus of recommendations showing medians and interquartile ranges (IQR) are displayed in Fig. 1. All recommendations achieved medians that indicated high to moderate consensus on their importance. The maximum median level of importance was attributed to two macro level Nos. 5 and 12 (see Table 2 and Fig. 1). Ten recommendations across macro, meso and micro categories had medians of 8, and a further 11 recommendations across all categories had medians of 7. There was a greater diversity in responses to several macro level statements (Nos. 4, 13, 15, 16, 17) and one meso level statement (No. 2) with wider IQRs. This suggests less agreement with the importance of these recommendations. In summary, higher priority recommendations related to education, leadership and policy-making. Medium priority recommendations focused on funding and relationship-building, and low priority recommendations focused on improving systems and infrastructure.

We present a content analysis highlighting the key domains.

### Education

Experts regarded education as a priority. It was considered that palliative care should be integrated into mandatory education for undergraduate medical, health and social care professionals (No. 12). Also, important although lower ranked was the inclusion of integrated palliative care in the continuing professional development of health and social care professionals (No. 13). This indicates that experts regard education about integrated palliative care to be a higher priority for those at the beginning of their health and social care careers.

### Awareness raising

Experts felt that raising public awareness of palliative care and its integration with healthcare was a key priority (No. 23). However, raising awareness of palliative care amongst senior managers was viewed as a lesser priority (No. 20). Experts, it seems, consider the publics’ lack of awareness as a greater challenge to integrated palliative care. Furthermore, it was considered that greater clarification of language and terms used to describe integrated palliative care and associated services was needed (No. 10) suggesting that the lack of public awareness may be affected by the complexity of language and terms used within this context, and a lack of professional agreement about terminology used.

### Leadership

Strong leadership to advocate for integrated palliative care was also considered a priority by experts (No. 16). However, the development of leadership skills was not prioritised (No. 17). The need to identify ‘champions’ and succession plan for these people was seen as more important than merely offering leadership skills training.

### Policy making

Experts shared a consensus about the need to include integrated palliative care at policy level. It was noted that palliative care for cancer patients is well established however the provision for patients with other conditions is often less accessible. Experts thus prioritised the extending of national palliative care regulations and policies to all patients with palliative care needs, not just those with cancer (No. 5). In addition to this, it was felt that palliative care should be integrated into all national policies relating to specific diseases (No. 15).

### Ensuring quality of care

Experts strongly prioritised the need for ensuring quality of services through auditing and benchmarking. However, emphasis was placed on the development of tools to be able to assess outcomes (No. 1), suggesting that there is a gap in this area. The practices of auditing and benchmarking these outcomes (No. 22) were given less priority suggesting that benchmarking is not relevant without reliable data. Thus, the development of assessment tools that monitor integration has greater urgency.

### Building relationships

It was observed that for experts the building of relationships was considered important for integrating palliative care, but was not considered a top priority. Within this, there was greater emphasis placed on developing alliances within and between health care sectors care (No. 11) than on exploring opportunities to establish informal relationships (No. 6) [8].

### Improving systems and infrastructure

Although considered important, recommendations relating to the development of better systems and infrastructure were not highly prioritised by experts. For example, the creation of a needs-based referral system to guide timely referrals to integrated care was ranked 22nd. Similarly, the development of an information hub, (online or a face-to-face central resource for the coordination of information exchange), with a care co-ordination team to contribute to the integration of palliative care services across geographical areas was perceived as less important (No. 2). Nonetheless, experts did prioritise the digital transfer of information within and across different palliative care services and generalist services such as GPs, community nurses and hospital teams (No. 3). Experts also felt more strongly about establishing a single point of contact for integrated palliative care at local level (No. 19) indicating a more urgent need to organise care clearly. Experts also favoured the introduction of a clinical protocol to ensure integration of palliative care services for patients and families regardless of the setting where they are treated (No. 7).

In relation to maintaining a work life balance for health care practitioners, providing adequate out-of-hours integrated palliative care was regarded as lower priority (No. 8). This may suggest that concerns about work/life balance for integrated care practitioners are less visible to experts.

### Funding and finance

Experts’ ranking of the recommendations also highlighted concerns around the funding of integrated palliative care. They prioritised the importance of establishing new and creative ways of securing resources in order to support the infrastructure of palliative care (No. 4). Experts also favoured national level strategic lobbying as a way to develop and fund better integrated palliative care (No. 9). In relation to medication however, the need for readily available and affordable essential medicines for integrated palliative care (No. 21) was ranked lower suggesting that experts’ concerns around funding are macro-level related [3].

## Discussion

### Main findings

International experts are uniquely positioned to provide insights into what recommendations are needed to strengthen, and what are priorities to implement, integrated palliative care. They have extensive experience of healthcare systems and are regarded as opinion leaders. Experts generated 23 recommendations, most referring to macro level organisation, perhaps reflecting their policy orientation and international operational interests. We also present novel data on their priorities for implementation of integrated palliative care, where education, leadership, assessment, communication using electronic systems and clear terminology are regarded as highly important.

While there is increasing recognition of the importance of integrated palliative care [2, 6, 9], there is little guidance on how integrated palliative care can be operationalised and in the contexts of constrained healthcare budgets, what should be prioritised for implementation. The results of this study shed light on which topics international leaders regard as priorities although perhaps with some bias towards macro goals as acknowledged above.

Previously, the WHO advocated a four-component model as a foundation for an international public health approach to palliative care [16]. The components comprise of: availability of essential medicines, especially access to opioids, education and training in core palliative care principles and skills for health professionals to build work force capacity, national health policies and strategic plans that incorporate palliative care and earmark resources, and implementation of a range of services [16]. While recommendations were generated in all four areas, there was an apparent shift in prioritisation to education and policy domains. International evidence suggests that the inclusion of palliative medicine in medical curricula remains limited in most countries [17, 18].

Experts also prioritised the implementation of national policies including integrated palliative care. However, the development of specific palliative care policies remains very much underdeveloped despite the growing interest from policy makers and governments, and endorsement by the WHA [2]. Most countries have not integrated palliative care in their national legislation, very few have produced specific palliative care national plans, and a minority (37%) of countries have an operational national policy for non-communicable diseases that includes palliative care [19].

### Strengths and limitations

Our study eliciting the recommendations and priorities for integrated palliative care from international experts addresses an important gap in the literature. This is the first comprehensive workshop designed to bringing together a range of expertise to discuss this topic but should be interpreted considering several limitations. The TEC methods were appropriate and feasible, but Delphi methodology may have been stronger. The results indicate the views of a small sample of selected international experts, predominantly Europeans, who were publishing on the topic of integrated palliative care and/or practicing clinically or were senior leaders of national or international organisations. We only included one national volunteer organisation. We acknowledge the critique of experts potentially being a biased resource [20] and a forthcoming paper will explore the views of clinicians. The selection of experts from high-income countries may account for a lack of prioritisation of certain topics such as opioid access, which are restricted in many low and middle-income countries [3]. Further research is required that explores perspectives of others including physicians and service users.

### Implications for policy and practice

Our findings suggest that amongst international experts there was good agreement on the importance of recommendations for integrated palliative care. The prioritisation of mandatory education for all health and social care undergraduates accords with the international literature but evidence suggests that it is long way from being universally included in medical and nursing curricula. Increasing the provision of integrated palliative care to non-cancer patients is warranted. Policy implications for greater inclusion of these topics are urgently required in national health plans. However, the results also raise questions about how priorities are identified and the influence of different stakeholders, especially those from wealthier countries.

## Conclusions

Our findings suggest that amongst a group of international experts there was overall good agreement on the importance of recommendations for integrated palliative care. Understanding expert’s priorities is important for investment of resources and can guide practice, policymaking and future research.

## Abbreviations

COPD: Chronic Obstructive Pulmonary Disease
INGOs: International non-governmental organisations
InSup-C: Integrated palliative care in cancer and chronic conditions
IQR: Interquartile ranges
NGOs: Non-governmental organisations
TEC: Transparent Expert Consultation
UK: United Kingdom
USA: United States of America
WHA: World Health Assembly
WHO: World Health Organisation
